# Nociceptive adenosine A_2A_ receptor on trigeminal nerves orchestrates CGRP release to regulate the progression of oral squamous cell carcinoma

**DOI:** 10.1038/s41368-024-00308-w

**Published:** 2024-06-18

**Authors:** Lanxin Jiang, Ying Zhou, Shijie Tang, Dan Yang, Yixin Zhang, Jiuge Zhang, Fan Yang, Tong Zhou, Xiaoqiang Xia, Qianming Chen, Lu Jiang, Yuchen Jiang, Xiaodong Feng

**Affiliations:** 1 State Key Laboratory of Oral Diseases & National Center for Stomatology & National Clinical Research Center for Oral Diseases & Research Unit of Oral Carcinogenesis and Management, Chinese Academy of Medical Sciences, West China Hospital of Stomatology, Sichuan University, Chengdu, China; 2 Key Laboratory of Oral Biomedical Research of Zhejiang Province, Affiliated Stomatology Hospital, Zhejiang University School of Stomatology, Hangzhou, China

**Keywords:** Cancer microenvironment, Mechanisms of disease

## Abstract

Oral squamous cell carcinoma (OSCC) associated pain commonly predicts adverse events among patients. This clinical feature indicates the engagement of nociceptors on sensory neurons during the development of malignancy. However, it is yet to be determined if targeting oncometabolite-associated nociception processes can hinder OSCC progression. In this study, we reported that nociceptive endings infiltrating both clinical samples and mouse tumor xenografts were associated with poorer clinical outcomes and drove tumor progression in vivo, as evidenced by clinical tissue microarray analysis and murine lingual denervation. We observed that the OSCC microenvironment was characteristic of excessive adenosine due to CD73 upregulation which negatively predicted clinical outcomes in the TCGA-HNSC patient cohort. Notably, such adenosine concentrative OSCC niche was associated with the stimulation of adenosine A_2A_ receptor (A_2A_R) on trigeminal ganglia. Antagonism of trigeminal A_2A_R with a selective A_2A_R inhibitor SCH58261 resulted in impeded OSCC growth in vivo. We showed that trigeminal A_2A_R overstimulation in OSCC xenograft did not entail any changes in the transcription level of CGRP in trigeminal ganglia but significantly triggered the release of CGRP, an effect counteracted by SCH58261. We further demonstrated the pro-tumor effect of CGRP by feeding mice with the clinically approved CGRP receptor antagonist rimegepant which inhibited the activation of ERK and YAP. Finally, we diminished the impact of CGRP on OSCC with istradefylline, a clinically available drug that targets neuronal A_2A_R. Therefore, we established trigeminal A_2A_R-mediated CGRP release as a promising druggable circuit in OSCC treatment.

## Introduction

Cancer-associated pain has emerged as an independent predictor for dire clinical prognosis.^1^ In oral squamous cell carcinoma (OSCC), patients with a higher stage of OSCC are more likely to report pain during their initial visit.^2^ Those who complain about increasing pain after completion of treatment are prone to suffer from regional reoccurrence and poorer survival.^3^ These clinical manifestations are in part due to the hyper-innervation nature of OSCC, an essential anatomical niche that features a major portion of sensory components, such as those originating from the trigeminal ganglion.^4^ The sensation of pain requires that OSCC-associated sensory nerve endings detect noxious signals within the tumor microenvironment. During the process of nociception, the sensory neurons synthesize and release neurotransmitters, which induce sensitization both peripherally to orchestrate local responses and centrally to alter mental perception.^5^ However, few studies focused on answering whether sensory neurons actively participate in the development of malignancy in response to nociceptive metabolites generated by OSCC.

Peripheral sensory nerve endings could be agitated by a variety of nociceptive metabolites, including adenosine.^6^ Extracellular adenosine mainly originates from ATP released from stressed or dying cells, catalyzed by ectoenzymes such as CD73 (encoded by *NT5E*) which is upregulated under hypoxic conditions.^7^ Adenosine-associated nociception is mediated by four receptors expressed on sensory neurons, namely A_1_R A_2A_R, A_2B_R, and A_3_R.^8^ Previous studies have extensively illustrated how adenosine worsened OSCC progression by suppressing anti-tumor immunity or directly regulating cancer cell behaviors.^9^ However, few studies have tackled whether and how adenosine interacts with sensory neurons to support OSCC growth, and if the molecular events underlying adenosine-associated nociception process can be pharmaceutically targeted as a regulator of OSCC progression.

Here, we reported that nociceptors innervated OSCC were immersed in an adenosine-concentrated environment which was associated with poorer clinical outcomes. Surgical denervation and selective inhibition of trigeminal A_2A_R with SCH58261 impeded OSCC growth. Administration of SCH58261 restricted the transient release of calcitonin gene-related peptide (CGRP) without affecting its transcription level in trigeminal ganglion. We then demonstrated that antagonism of the CGRP receptor with rimegepant, a drug approved for migraine treatment, could slow down tumor growth in vivo. Finally, we exploited istradefylline (KW6002), a clinically available A_2A_R antagonist as an agent to treat mice with tumor xenograft which exhibited an anti-proliferative effect while lowering serum CGRP level. Taken together, these findings underlined the growth-promoting role of trigeminal A_2A_R in the progression of OSCC and further established the trigeminal A_2A_R-CGRP circuit as a potential therapeutic target.

## Results

### Nociceptive nerves infiltrate the OSCC microenvironment enriched in adenosine

**Fig. 1:**
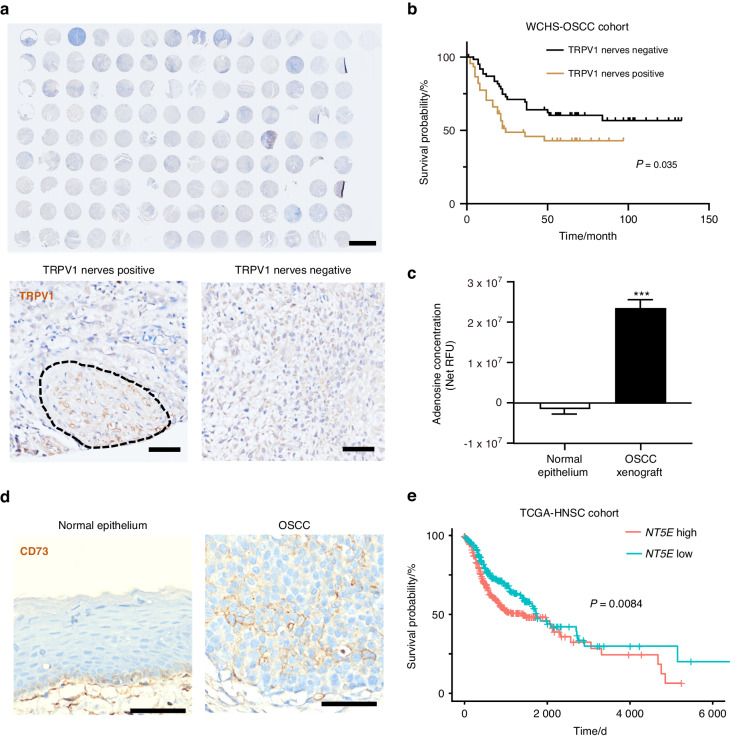
Presence of nociceptive nerves in adenosine-concentrated OSCC. **a** The upper panel presents the broad view of immunostaining of TRPV1 in the patient cohort collected by West China Hospital of Stomatology (WCHS). Scale bar: 2 mm. The lower panel denotes representative samples with and without TRPV1 neuronal infiltration (‘TRPV1 positive’ and ‘TRPV1 negative’). Scale bar: 50 µm. **b** Kaplan–Meier plot delineating survival probability for WCHS-OSCC patients stratified against TRPV1 neuron infiltration in their tumors, *n* = 111 patients. **c** Comparison of adenosine concentration in normal epithelium and HSC3 xenograft of mice, represented as net relative fluorescence unit (RFU). *n* = 3 mice. **d** Immunostaining of CD73 in human normal epithelium and human OSCC cells. Scale bars, 50 μm. **e** Kaplan–Meier plot delineating survival probability for TCGA-HNSC patients stratified against *NT5E* expression in their tumors. *NT5E*-high and *NT5E*-low groups are defined as above or below the median of *NT5E* expression. The TCGA-HNSC cohort database analyzed was updated to March 29th, 2023, *n* = 518 patients. Statistical analysis was conducted using unpaired Student’s *t*-test (**c**) and Log-rank test (**b**, **e**)

Pain or other sensory abnormalities are frequently reported by OSCC patients.^10^ Accordingly, we found that nociceptive TRPV1^+^ neurons infiltrate tumor mass among OSCC patients (Supplementary Fig. 1a). Of note, the infiltration of nociceptors was associated with worse survival probability, lymph node metastasis, and higher clinical stage among the patient cohort we collected (Fig. 1a, b and Table 1). Additionally, the rate of nociceptive nerve infiltration almost doubled in the T4 tumor stage compared to that of the lower tumor stage (Table 1). To seek the potential molecular that could sensitize cancer-associated nociceptive nerve endings, we established an OSCC orthotopic xenograft in mice which could induce prominent nociceptive phenotypes.^4^ Notably, we detected a significantly higher amount of adenosine in TRPV1-positive neuron innervated OSCC xenograft, compared with normal epithelium (Fig. 1c and Supplementary Fig. 1a). As CD73 represents the essential catalyzer to produce adenosine, we analyzed the expression of CD73 in OSCC clinical samples. While CD73 was invariably absent from normal non-basal epithelial cells, it was exclusively overexpressed on OSCC tumorous cells (Fig. 1d). On top of that, we noted that higher levels of CD73 expression predicted worse overall survival among HNSC patients of the TCGA cohort (Fig. 1e and Supplementary Fig. 1b). Taken together, these findings suggest that nociceptive nerve endings are immersed in an adenosine concentrative OSCC microenvironment.

**Table 1:** Association between TRPV1^+^ nerve infiltration and clinical grade, T staging, and lymph node metastasis of OSCC patients

Variables	TRPV1^+^ nerve negative/number of cases (%)	TRPV1^+^ nerve positive/number of cases (%)	*P*-value
Clinical grade			0.022
Grade1	9 (69%)	4 (31%)	
Grade2	25 (71%)	10 (29%)	
Grade3	18 (49%)	19 (51%)	
Grade4	13 (45%)	16 (55%)	
T stage			0.105
T1	12 (60%)	8 (40%)	
T2	35 (63%)	21 (38%)	
T3	14 (70%)	6 (30%)	
T4	6 (32%)	13 (68%)	
Lymph node metastasis			0.023
Negative	43 (68%)	20 (32%)	
Positive	24 (46%)	28 (54%)	

The association between clinical staging or T staging and TRPV1^+^ nerve infiltration was examined using the Cochran-Armitage trend test, while the relationship between lymph node metastasis and TRPV1^+^ nerve infiltration was assessed using Fisher’s exact test

### Antagonism of trigeminal A_2A_R in vivo stifled the growth of OSCC

**Fig. 2:**
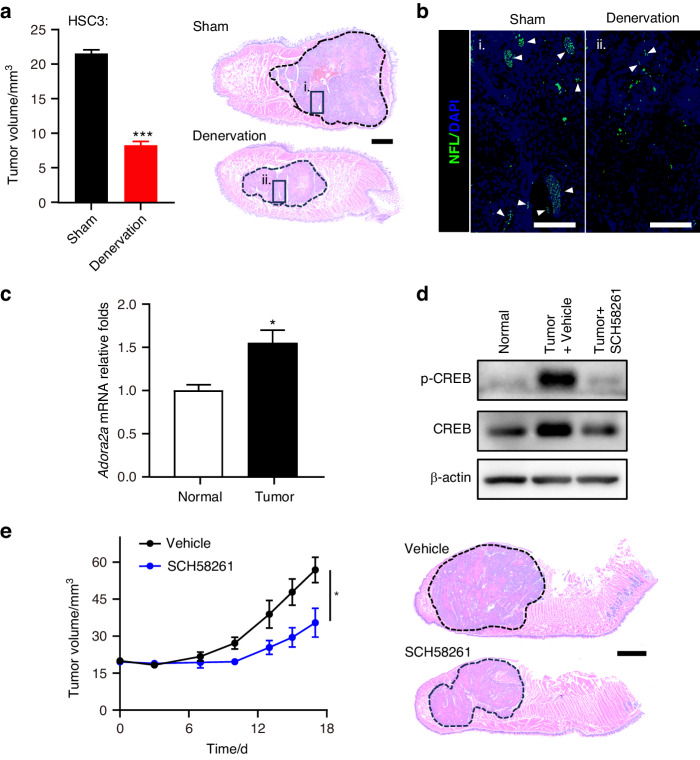
Surgical denervation and antagonism of trigeminal A_2A_R with SCH58261 inhibited OSCC growth. **a** Tumor volume of HSC3 xenografts in mice undergoing sham surgery or lingual nerve denervation, *n* = 4 mice (Left panel). Representative HE images of HSC3 xenograft (circled) in mice undergoing sham surgery or lingual denervation (Right panel). Scale bar, 500 µm. **b** Immunofluorescent staining of neurofilament light chain (NFL, arrowheads) in framed area of **a**. Scale bars, 100 µm. **c** Relative mRNA expression of *Adora2a* in trigeminal ganglia of healthy mice or mice carrying HSC3 tumor xenograft, *n* = 3 mice. **d** Phosphorylation of CREB and total CREB in trigeminal ganglia of healthy mice, mice carrying HSC3 tumor xenograft, and tumor-bearing mice treated with 5 mg/kg SCH58261 for 3 h. **e** In vivo growth of HSC3 xenograft in nontreated mice and those treated with 5 mg/kg SCH58261, *n* = 6 mice. (Left panel) Representative HE images of HSC3 xenografts in mice treated with vehicle or 5 mg/kg SCH58261 (Right panel). Scale bar, 1 mm. Statistical analysis was conducted using unpaired Student’s t-test (**a**, **c**, **e**)

To test whether nociceptive innervation is detrimental to the progression of OSCC, we surgically deprived mice of lingual nerve, the primary afferent from TG that innervates the tongue area. This procedure that transected most activated nociceptors in the tumor microenvironment induced a significant reduction in different types of OSCC xenografts (Fig. 2a, b and Supplementary Fig. 2a, b). Given that adenosine signaling is negatively associated with disease outcome,^11^ we then searched for the receptor on trigeminal nerve endings that might mediate the pro-tumor effect of adenosine. It has been suggested that A_2A_R on periphery sensory neurons mediates the pronociceptive effect following adenosine stimulation.^6^ Therefore, we hypothesized that trigeminal A_2A_R was potentially stimulated by adenosine and that the antagonism of trigeminal A_2A_R could partially counteract the growth-promoting role of trigeminal nerve endings. To confirm this hypothesis, we investigated trigeminal ganglia (TG) that innervate tumor inoculation side of the tongue, as they provide direct evidence of how nociceptors interact with OSCC at both transcription and protein levels. Compared with TG collected from healthy controls, the mRNA level of *Adora2a* increased in those of tumor-bearing mice (Fig. 2c). On top of that, the phosphorylation of CREB in TG, the main downstream target of A_2A_R mediated Gα_s_ signaling,^12^ was prominently upregulated by OSCC, while SCH58261, a selective A_2A_R inhibitor, reversed this effect (Fig. 2d). We then hypothesized that the hyperactived trigeminal A_2A_R might contribute to the progression of OSCC. Intriguingly, treatment with SCH58261 at a dose sufficient to block A_2A_R signaling in TG (Fig. 2d) impeded the growth of tumor xenograft (Fig. 2e). Taken together, these data highlight that trigeminal A_2A_R was activated in OSCC and could potentially contribute to tumor growth.

### Trigeminal A_2A_R mediates CGRP release from sensory neuron

**Fig. 3:**
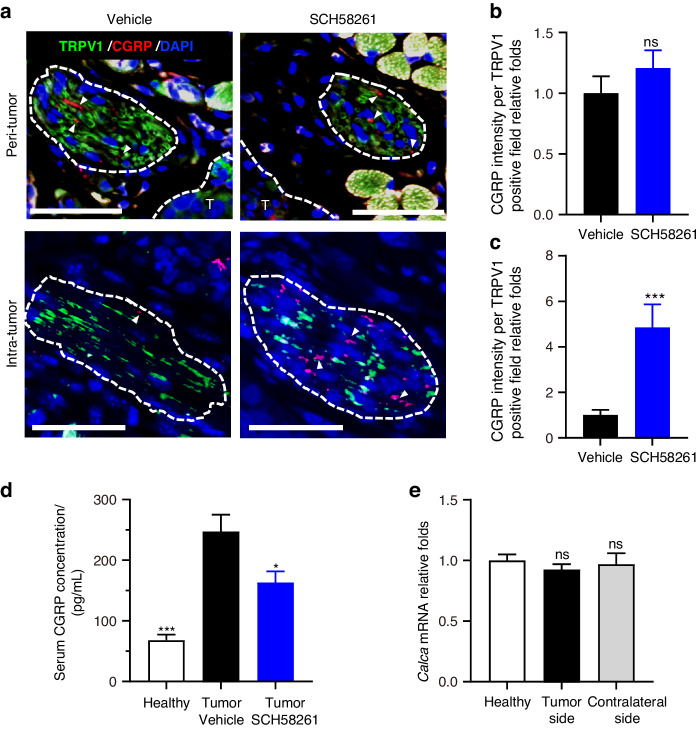
SCH58261 blocked CGRP release from nociceptive endings**. a** Represented immunostaining of CGRP (red, arrowheads) in TRPV1^+^ neuronal niche (green, circled). The curves in the upper panels delineated the tumor area (T, blue). Scale bars, 50 μm. **b** Comparison of CGRP immunofluorescence intensity of each TRPV1-positive field (1 000 µm^2^) in the peri-tumor area represented, *n* = 16 random fields from 4 mice. **c** Comparison of CGRP immunofluorescence intensity of each TRPV1-positive field (1 000 µm^2^) in intra-tumor area. *n* = 16 random fields from 4 mice. **d** CGRP level in serum from healthy mice, tumor-carrying mice with or without 5 mg/kg SCH58261 treatment. *n* = 5 mice. **e** Relative transcription level of *Calca* in trigeminal ganglia of healthy mice, ipsilateral or contralateral side of tumor inoculation site, *n* = 3 mice. Statistical analysis was conducted using unpaired Student’s *t*-test (**b**, **c**, **d**, **e**)

Next, we investigated how trigeminal ganglia reacted to A_2A_R activation which led to tumor growth. External stimulation can uniquely trigger the instant release of neurotransmitters from vesicles docking on the nerve-ending membrane.^13^ Prior studies suggest that the secretion of CGRP mediated by adenosine is contingent upon the interplay between A_1_R and A_2A_R. At elevated adenosine concentrations, A_2A_R activation occurs,^14,15^ which subsequently diminishes the affinity of A_1_R for adenosine.^16,17^ This mechanism effectively antagonizes A_1_R mediated suppression of CGRP secretion.^18^ Given that CGRP participates in adenosine-induced nociceptive events, we postulated that the stimulated A_2A_R on trigeminal nerve endings might orchestrate the release of CGRP within the OSCC microenvironment. We noted that following SCH58261 treatment, the CGRP signal did not alter in TRPV1^+^ nerves surrounding tumor xenograft (Fig. 3a, b), yet significantly enhanced in intra-tumoral nociceptors (Fig. 3a, c). Besides, A_2A_R inhibition significantly decreased the level of circulating CGRP in tumor-bearing mice (Fig. 3d). Note that the CGRP stained in nerve fibers was representative of the CGRP unreleased from nociceptive neurons, while the perturbances in circulating CGRP captured the pool of CGRP released. Therefore, these data suggested that trigeminal A_2A_R blockage with SCH58261 might restrain the release of CGRP from the proportion of sensory nerve fibers infiltrating OSCC, which could contribute to the reduction in circulating CGRP level. Although other in vitro studies showed that TG co-cultured with OSCC cells transcriptionally upregulated CGRP,^19^ we did not notice any significant change of CGRP transcription in TG of either side (Fig. 3e). Taken together, these findings suggest that overstimulation of trigeminal A_2A_R induced a robust release of CGRP in OSCC microenvironment.

### CGRP receptor antagonism induced anti-proliferation effects in vivo

**Fig. 4:**
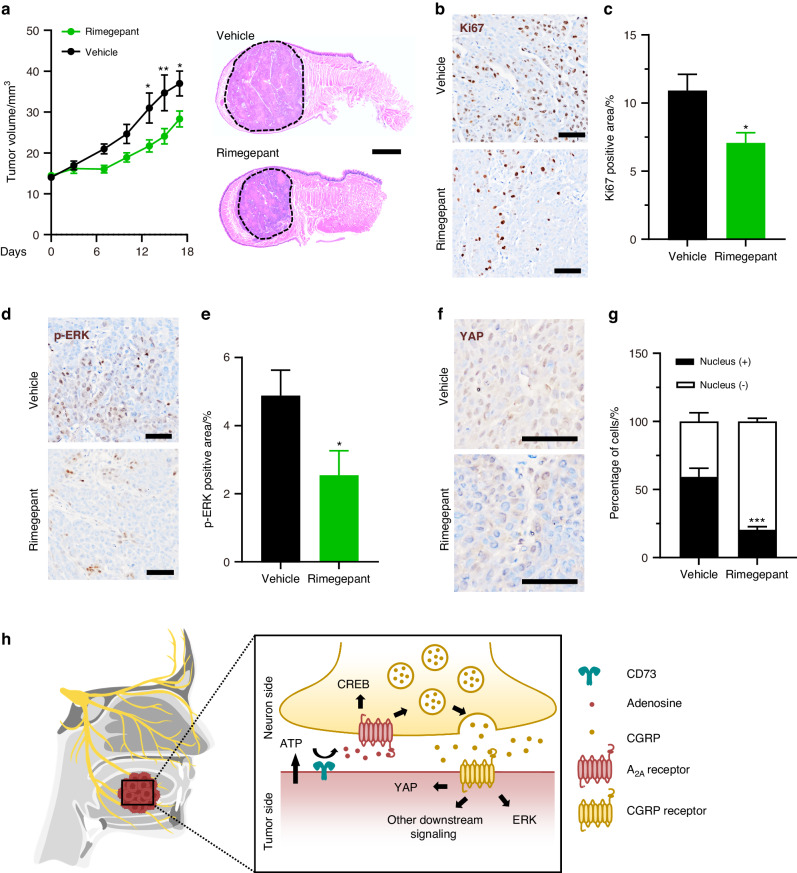
CGRP receptor antagonist inhibited phosphorylation of ERK and YAP in OSCC xenograft. **a** In vivo growth of HSC3 xenograft in nontreated mice and those treated with 20 mg/kg rimegepant, *n* = 6 mice. (Left panel). Representative HE images of HSC3 xenografts in mice treated with vehicle or 20 mg/kg rimegepant. (Right panel). Scale bar, 1 mm. **b** Immunostaining of Ki67 in tumor xenograft in nontreated or treated group. Scale bars, 50 μm. **c** Quantification of Ki67-positive area in tumor xenograft of the nontreated or treated group, *n* = 15 random fields from 5 mice. **d** Immunostaining of p-ERK in tumor xenograft of nontreated or treated group. Scale bars, 50 μm. **e** Quantification of p-ERK-positive area in tumor xenograft of the nontreated or treated group, *n* = 15 random fields from 5 mice. **f** Immunostaining of YAP in tumor xenograft of nontreated or treated group. Scale bars, 50 μm. **g** Quantification of the percentage of YAP nuclear-positive cells of the nontreated or treated group, *n* = 9 random fields from 3 mice. **h** Simplified illustration of trigeminal A_2A_R mediated CGRP release that activates ERK and YAP in tumor cells. Statistical analysis was conducted using two-way ANOVA with Bonferroni post hoc test (**a**) and unpaired Student’s *t*-test (**c**, **e**, **g**)

Along with a diminishment in Ki67-positive cells (Supplementary Fig. 3a, b), we noticed that trigeminal A_2A_R antagonism blocked ERK activation (Supplementary Fig. 3c, d) and restrained the nuclear localization of YAP in OSCC xenograft (Supplementary Fig. 3e, f). Given that CGRP has been identified as a mitogen that could act through activating ERK and YAP under physiological conditions,^20,21^ we proceeded to investigate if the significant reduction in these proliferative signatures of OSCC was partially a consequence of trigeminal A_2A_R induced CGRP release, independent of other potential SCH58261 mediated effects. We fed tumor-bearing mice with rimegepant, a CGRP receptor antagonist which induced a significant reduction in tumor volume (Fig. 4a). Histological analysis verified this finding, showing a significantly lower proportion of Ki67-positive tumor cells in mice undergoing rimegepant treatment (Fig. 4b, c). On top of that, we found that ERK was deactivated in rimegepant-treated mice (Fig. 4d, e), with a significant downregulation in the proportion of YAP-positive nucleus (Fig. 4f, g). Taken together, these data indicate that the CGRP receptor mediates the activation of YAP and ERK that drive tumor growth (Fig. 4h).

### Trigeminal A_2A_R represents a therapeutic target in OSCC

**Fig. 5:**
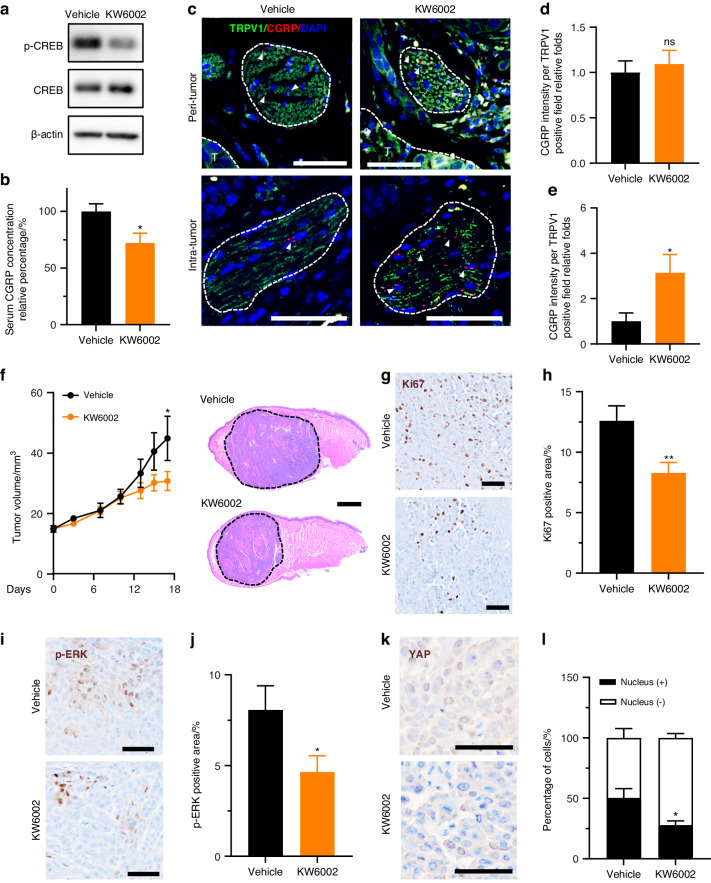
Istradefylline impeded OSCC growth by blocking trigeminal A_2A_R-induced CGRP release. **a** Phosphorylation of CREB and total CREB in trigeminal ganglia of mice carrying HSC3 tumor xenograft and tumor-bearing mice treated with 6 mg/kg KW6002 for 3 h. **b** Relative percentage of CGRP level in serum from tumor-carrying mice with or without 6 mg/kg KW6002 treatment. *n* = 6 mice. **c** Represented immunostaining of CGRP (red, arrowheads) in TRPV1^+^ neuronal niche (green, circled 1 000 µm^2^). The curves in the upper panels delineated the tumor area (T, blue). Scale bars, 50 μm. **d** Comparison of CGRP immunofluorescence intensity of each TRPV1-positive field (1 000 µm^2^) in the peri-tumor area represented, *n* = 9 random fields from 3 mice. **e** Comparison of CGRP immunofluorescence intensity of each TRPV1-positive field (1 000 µm^2^) in intra-tumor area. *n* = 9 random fields from 3 mice. **f** In vivo growth of HSC3 xenograft in nontreated mice and those treated with 6 mg/kg KW6002, *n* = 6 mice. (Left panel). Representative HE images of HSC3 xenografts in mice treated with vehicle or 6 mg/kg KW6002. Scale bar, 1 mm. (Right panel). **g** Immunostaining of Ki67 in tumor xenograft in nontreated or treated group. Scale bars, 50 μm. **h** Quantification of Ki67-positive area in tumor xenograft in the nontreated or treated group, *n* = 15 random fields from 5 mice. **i** Immunostaining of p-ERK in tumor xenograft of the nontreated or treated group. Scale bars, 50 μm. **j** Quantification of p-ERK-positive area in tumor xenograft of the nontreated or treated group, *n* = 15 random fields from 5 mice. **k** Immunostaining of YAP in tumor xenograft of nontreated or treated group. Scale bars, 50 μm. **l** Quantification of the percentage of YAP nuclear-positive cells of nontreated or treated group, *n* = 9 random fields from 3 mice. Statistical analysis was conducted using unpaired Student’s *t*-test (**b**, **d**, **e**, **h**, **j**, **l**) and two-way ANOVA with Bonferroni post hoc test (**f**)

At this point, we concluded that trigeminal A_2A_R activated by an adenosine-intensive OSCC microenvironment triggered the release of pronociceptive neuropeptide which in turn potently fueled OSCC growth. We next considered if istradefylline (KW6002), a clinically available A_2A_R antagonist approved for treating Parkinson’s disease with less selectivity and affinity to A_2A_R than SCH58261^22^ could recapitulate this crosstalk between trigeminal nerve endings and OSCC. We intraperitoneally injected mice with KW6002 at a dose that significantly downregulated phosphorylated CREB levels in TG (Fig. 5a). At terminal point, serum level of CGRP in mice treated with KW6002 dropped about 30% compared with their control littermates (Fig. 5b), while immunofluorescent staining showed that KW6002 curtailed the release of CGRP from intra-tumoral nociceptive nerve endings (Fig. 5c–e). We noticed that KW6002 significantly hindered the proliferative ability of OSCC cells in vivo (Fig. 5f–h). Similar to CGRP receptor antagonism, trigeminal A_2A_R inhibition also contained ERK and YAP activation (Fig. 5i–l). Taken together, these data confirmed our belief that trigeminal A_2A_R could be clinically targeted among OSCC patients with KW6002.

## Discussion

Cancer pain is frequently linked to adverse clinical outcomes in OSCC patients,^2,3,23^ indicating a malignant role of pain in the development of OSCC. While the perception of pain relies on an individual’s sensitivity to noxious stimuli, pain is invariably predicated on nociception which operates independently of the central nervous system.^5^ Therefore, we reasoned that nociception should facilitate OSCC. In our study, we pinpointed nociceptive A_2A_R as a critical contributor to OSCC progression. In particular, adenosine generated from orthotopically engrafted OSCC induced the firing of trigeminal A_2A_R, leading to intra-tumoral neurogenic release of CGRP which promotes tumor growth through activation of ERK and YAP.

Despite the growing body of clinical trials assessing A_2A_R inhibitors across various other cancers, such as renal cell cancer and non-small cell lung cancer,^24,25^ a gap in preclinical evidence has notably limited the examination of their efficacy in OSCC contexts. The current literature has rigorously established how adaptive immunity associated with A_2A_R promotes head and neck cancer.^26–28^ However, it has been neglected that a high amount of adenosine can also trigger neuronal A_2A_R,^6,29^ a potential contributor to OSCC progression. Our observations of the oral environment’s ‘sensitive’ nature, characterized by a rich sensory neuron network responsive to stimuli,^4^ led us to hypothesize a significant, yet underexplored, role for trigeminal nerve endings in OSCC progression. To sidestep the impact of the adaptive immune system on OSCC, we utilized the Balb/c nu/nu mouse model. Our study demonstrated that trigeminal A_2A_R was a targetable sensor that contributed to tumor growth. In response to excessive adenosine produced by OSCC xenograft, trigeminal A_2A_R was transcriptionally upregulated and CREB, the downstream of A_2A_R mediated signaling, was hyperactivated, although a genetic model should be employed to further determine if A_2A_R on trigeminal ganglia was exclusively triggered by adenosine in OSCC. Trigeminal A_2A_R also contributed to the progression of OSCC, as the potent A_2A_R inhibitor SCH58261 impeded tumor growth. Moreover, our findings highlight a potentially critical criterion for patient selection in clinical trials: the presence of tumors positive for CD73 staining and elevated serum CGRP levels, which may indicate an active A_2A_R-CGRP circuit and, by extension, identify patients who could benefit from A_2A_R inhibitor therapy.

The neuropeptide CGRP is a major participant in nocifensive events following neuron stimulation.^30^ Of note, the contribution of CGRP to cancer development has recently come into spotlight.^19,31,32^ However, how OSCC affects the dynamics of CGRP still remains controversial. Of note, one study employed an in vitro co-culture model to study the crosstalk between cancer cells and neurons. It revealed that the transcription of CGRP in trigeminal ganglia was upregulated when co-cultured with OSCC cells.^19^ However, another study found that the transcription activities associated with pain signaling in trigeminal ganglia were significantly downregulated when the neurons were cultured in media containing extracellular vesicles from OSCC.^33^ Given the polarized distribution of neurons where the soma and synaptic terminal are functionally diversified,^34^ we postulated that OSCC-mediated changes in CGRP activities do not necessarily involve regulation of gene transcription in the soma. Indeed, we noticed that compared with healthy controls, OSCC triggered a release of CGRP but did not affect the transcription level of *Calca* in trigeminal ganglia in vivo. More importantly, the antagonism of trigeminal A_2A_R with SCH58261 contained the release of CGRP, which was at least partially due to the restriction of CGRP release from nociceptive nerves within OSCC tumor mass. This finding was consistent with the fact that GPCR signaling can mediate the gating of calcium channels on neurons, which controls the fast excitation-secretion coupling of neurotransmitters stored in axon terminals.^13^

Current research has addressed that MAPK signaling is essential in CGRP receptor-mediated signaling,^19,35^ yet there still lacks a more comprehensive description of signaling pathways downstream of CGRP receptor in OSCC. CGRP receptor functions through a complex involving a dimerized RAMP1/CLR cross membrane complex and an intracellular receptor component protein (RCP), which fine-tunes the signals relayed by CLR.^36^ Interestingly, CGRPR activation is predicated on a sequential engagement of CGRP with the receptor, requiring the c-terminal of CGRP to engage with the RAMP1-CLR interface before the N-terminal engages with CLR’s loop and helix.^37^ CLR is a GPCR coupled to diverse Gα proteins, including Gα_q_.^36^ It has been well established that the activation of GPCRs could regulate YAP,^38^ a crucial element that reportedly contributes to OSCC development downstream of RhoA.^39–41^ Furthermore, transgenic mice with RAMP1 knockout demonstrate impaired liver regeneration capacity with decreased nuclei localization of YAP.^42^ In this study, we noticed a significant reduction in the phosphorylation level of ERK and YAP activation in OSCC cells following CGRP receptor antagonism in vivo, thereby sustaining YAP as a novel effector downstream of CGRP receptor activation independent of the MAPK signaling. Given the critical role of ERK and YAP per se in tumor development,^43,44^ we believe our results encourage further investigation of whether a combination of FDA-approved MEK inhibitors (trametinib, cobimetinib, binimetinib) and agents that inhibit YAP activation is effective in treating OSCC.^45^

Provided that surgical deprivation of sensory neurons can halt OSCC growth in this study, cutting off the crosstalk between nociceptors and cancer cells should be a viable targeted strategy in designing OSCC treatment. However, there is still a lack of definitive clinical evidence that supports the efficacy of analgesics or adjuvants in hindering OSCC growth. For example, the consumption of aspirin or nonsteroidal anti-inflammatory drugs, two nonopioid analgesics, is not necessarily associated with positive clinical outcomes of OSCC.^46,47^ More concerning is the fact that opioid usage, a common prescription for treating chronic cancer pain, is linked to decreased survival,^48^ along with grave complications such as emesis, constipation, and side effects on the central nervous system.^49^ Therefore, anti-cancer therapies that target the pain process should be based on a detailed breakdown of how nociceptive events mediate OSCC growth. Emerging evidence has delineated one pool of OSCC-derived molecules that agitate nociceptors, such as endothelin-1, ATP, NGF, and TRP-activating lipids,^19,50^ and another pool of neurotransmitters released from sensory neurons like noradrenaline.^33^ However, drugs designed for these targets are either yet to be clinically approved or lack clinical trials that support their efficacy in treating OSCC.^51^ Therefore, to promote rapid clinical translation, it is imperative to examine if there is any clinically available drug that can potentially break the nerve-cancer crosstalk to inhibit OSCC growth.

According to our proposed trigeminal A_2A_R-CGRP regulatory circuit, we established two potential druggable targets: the A_2A_R receptor and CGRP. While there is only one FDA-approved A_2A_R antagonist (istradefylline),^52^ several approved drugs are available for targeting CGRP or its receptor, including four monoclonal antibodies (erenumab, fremanezumab, galcanezumab, eptinezumab) and three “gepant” antagonists (ubrogepant, atogepant, rimegepant).^53^ Nevertheless, we chose rimegepant in our preclinical model owing to its convenient administration route,^53^ rapid onset of pharmacological action,^54,55^ sustained efficacy,^54,55^ and minimal adverse effects,^56^ compared with other candidates. Our in vivo results vividly support the efficacy of rimegepant or istradefylline in the OSCC-promoting trigeminal A_2A_R-CGRP circuit. Given the documented immunosuppressive effect associated with both A_2A_R and CGRP receptor,^31,57^ our findings suggest how a two-phase dynamic of neuro-immune interactions might impact OSCC in immunocompetent model animals. During the early stages of tumor development, the immune cells vigorously attack the tumor while producing pronociceptive cytokines such as IFNγ,^58^ thus triggering the release of immunosuppressive CGRP from nociceptive endings.^31^ As the tumor progresses, immune cells generate abundant adenosine,^27,59^ sufficient to trigger nociceptive A_2A_R-mediated secretion of CGRP, as our study implies, which further limits the function of immune cells. Therefore, further investigations are warranted to assess the potential synergistic effects and biosafety of the combined use of rimegepant and istradefylline in restoring both cancer immunosurveillance and alleviating cancer pain.

In summary, our research identified the trigeminal A_2A_R-mediated nociception process as a critical contributor to OSCC progression. Mechanistically, stimulated A_2A_R signaling in trigeminal ganglia was associated with increased levels of CGRP in mice bearing OSCC xenograft. Antagonism of A_2A_R decreased trigeminal release of CGRP which inhibited OSCC growth via suppressing ERK and YAP activation. In light of this trigeminal A_2A_R-CGRP circuit, we successfully repurposed rimegepant and KW6002, two clinically approved drugs, to treat OSCC in vivo.

## Materials and methods

### Clinical samples and data analysis

For immunostaining and clinical tissue microarray analysis, 122 OSCC patients receiving surgical operation were collected by West China Hospital of Stomatology under the approval of the Scientific and Ethical Committee of Sichuan University. Clinical features such as age, sex, primary tumor site, TNM stage, and survival duration were comprehensively documented. Patients were provided with written informed consent concerning the usage of their specimens in laboratory experiments. For survival analysis associated with TRPV1 neuron infiltration, a total of 117 patient samples with discernible tumor cells were included. For bioinformatics analysis, HNSC patient data were downloaded from the TCGA database. Kaplan–Meier survival analysis was conducted using R 4.3. The differentially expressed genes were analyzed through ‘edgeR’ and ‘limma’ packages. Log2 counts per million (CPM) value was calculated by ‘limma’ package through the ‘voom’ approach. The analysis and visualization of patient survival data were achieved through the ‘survival’ and ‘survminer’ packages.

### Animals

Male BALB/c nu/nu mice aged 6 weeks were purchased from Charles Rivers Laboratories. Mice were housed and underwent each experimental procedure in specific pathogen-free conditions in 12 h–12 h light–dark cycles at 24 °C and 50% humidity. All experimental procedures were approved by the Animal Ethics Committee of Sichuan University (Approval No. 20220304057) and conformed to ARRIVE Guidelines.

### Surgical lingual denervation

Mice were randomly assigned to sham surgery or lingual denervation group. The lingual transection was performed as previously described.^33^ The left chorda-lingual nerve was exposed between the masseter muscles and the anterior belly of the digastric. The nerve was carefully transected after being separated from the surrounding fascia. To minimize regeneration, the distal and proximal stumps were further resected in a 5 mm section. For sham surgery, the nerve was exposed as described before without being transected. Each surgery was performed in less than 10 min and no complications were recorded in the following week. One week after surgery, mice regained weight and were subjected to tumor inoculation.

### Drug administration

5 mg/kg SCH58261 (MCE HY-19533) was dissolved in a vehicle containing 2% DMSO, 40% PEG300 (MCE HY-Y0873), 4% Tween80 (MCE HY-Y1891), and 54% saline and was administered i.p. daily. 20 mg/kg rimegepant (TargetMol 1289023-67-1) was dissolved in carrier solution containing 2% DMSO, 40% PEG300, 5% Tween80 and 53% saline was fed p.o. daily. 6 mg/kg KW6002 (MCE HY-10888) was prepared in carrier solution consisting of 3% DMSO, 15% cremophor (MCE HY-Y1890), and 82% saline and was injected i.p. daily. In each independent experiment, mice were randomly assigned to vehicle or treatment groups four days post-tumor inoculation, and tumor volume was recorded from the first day of drug administration (Defined as Day 0).

### Cell line and murine tongue xenograft model

HSC3 and CAL27 were cultured in DMEM (HyClone) supplemented with 10% FBS (HyClone,). After cells reached a confluence level of 75%, they were digested by 0.25% trypsin (HyClone) and centrifuged. Tumor cells were then resuspended with ice-cold serum-free DMEM mixed thoroughly with growth factor reduced Matrigel (Corning, 354230) at a volume ratio of 3:1. Before the cell inoculation procedure, mice were anesthetized by 20 μL/g avertin and were irresponsive to toe pinch. Each mouse was submucosally injected 1.0 × 10^5^ tumor cells in 20 µL serum-free medium on the left middle portion of the tongue with a 26 g syringe needle and then placed on a heating pad until full recovery. Tumor size was monitored two to three times a week with a vernier caliper. To ensure accurate measurement, each tumor was measured three times, and the size was determined as the average of three individual volumes calculated as 0.5 × length × width^2^. In addition, the investigator who measured tumor volume was blinded to the treatment each mice received. The length or width was recorded and calculated separately by another investigator who was also blinded. No mice were excluded when comparing tumor volume between each group. The location of the cage was switched randomly after each measurement practice. Mice were weighted two to three times a week for the monitoring of overall well-being. All subjects were euthanized by cervical dislocation when a significant difference was reached in each experiment.

### Immunohistochemistry

All samples collected were fixed in 4% paraformaldehyde at 4 °C overnight before being embedded in paraffin and sectioned at 5 μm thickness. For fluorescent staining, antigen was retrieved through heated 10 mmol/L citric acid. Tissue sections were blocked in phosphate-buffered saline (PBS) containing 10% normal goat serum, 1% BSA, and 0.3% Triton-X for an hour and then incubated with diluted primary antibodies TRPV1 (Alomone labs ACC-030), CGRP (Abcam ab818870) and NFL (Millipore ab9568) at 4 °C overnight. Secondary fluorescence-conjugated antibodies (Invitrogen A11008, A11020) and DAPI (Vectorlab H-1200) were applied on the following day. For non-fluorescent staining, antigen was retrieved either through 1X Tris-EDTA (for CD73, p-ERK and YAP) or 10 mM citric acid (for TRPV1and Ki67) before incubated with corresponding primary antibody TRPV1 (Alomone labs ACC-030), CD73(CST 13160 S), p-ERK (CST 4370 S), Ki67 (HUABIO HA721115) and YAP (CST 14074) in blocking solution, followed by reaction enhancer solution and horseradish peroxidase (HRP)-conjugated secondary antibody (ORIGENE PV-9001). All non-fluorescent sections were counterstained with 4’,6-diamidino-2-phenylindole (DAB) (Gene tech GK600505). All tissue sections were scanned by Olympus VS200, and all images used in quantification analysis were captured by OlyVIA 4.1.

### Trigeminal ganglia protein analysis

Trigeminal ganglia were extracted and snap-frozen on liquid nitrogen. Tissue protein was thoroughly ultrasonicated in lysis buffer containing 98% SDS, 1% 1X protease inhibitor, and 1% Na_3_VO_4_ before being mixed with 1X loading buffer. For western blot analysis, tissue protein was electrophoresed on 10% polyacrylamide gels and transferred onto polyvinylidene difluoride membranes. The membranes were then briefly washed in 1X TBST, saturated with 5% skim milk for 30 min, and incubated with primary antibodies β-actin (Santa Cruz sc-69879), CREB (CST 9197S), p-CREB (CST 9198S) overnight at 4 °C. On the following day, HRP-conjugated secondary antibodies (ORIGENE ZB-2305, ZB-2301) were applied for 2 h at room temperature. The target protein was then visualized through chemiluminescent detection (Millipore WBKLS0500).

### Trigeminal ganglia mRNA expression analysis

Trigeminal ganglia were harvested and homogenized in TRIzol (Life technologies 15596026) with Precellys (Bertin technologies). Approximately 1 μg of RNA per ganglion was isolated with 1-bromo-3-chloropropane (Sigma-Aldrich 109-70-6). All RNA samples were reverse transcribed to DNA following the manufacturer’s protocols (TAKARA RR037A). Real-time qPCR was conducted with SYBR Green Master Mix (TAKARA RR820A) on LightCycler 96 (Roche). Primer sequences were listed in Supplementary Table 1.

### Adenosine measurement

Normal tongue epithelium and tumor mass were carefully isolated from muscle tissue. To minimize adenosine degradation, the tissue was protected in PBS buffer containing 1X protease inhibitor cocktail (Selleck 14002), 10 μmol/L EHNA (MCE HY-103160A), and 10 μmol/L ABT-702 (TargetMol T4668).^60^ The samples were homogenized with Precellys in the corresponding volume of buffer (weight to volume ratio = 1:9) and then centrifuged at 10 000 × *g* for 15 min at 4 °C. The supernatant was collected and immediately assayed following the manufacturer’s instructions (CELL BIOLABS MET-5090). The fluorescence signal was read by SpectraMax iD3 (MOLECULAR DEVICES). Net relative fluorescent unit (RFU) was the difference value that subtracted the sample well values without adenosine deaminase from those containing adenosine deaminase.

### CGRP measurement

Fresh venous blood from mice was collected and allowed to clot at 4 °C overnight. The samples were then centrifuged at 3 000 r/min for 15 min on the next day. The serum was immediately collected and assayed following the manufacturer’s protocol (Elabscience E-EL-M0215).

### Statistical analysis

All images were analyzed by ImageJ 1.52p. The statistical analysis was performed with GraphPad 9.5. The standard curve and absolute quantification of CGRP were processed by Origin 9.1. All data were presented as mean ± S.E.M. We gave 80% power for an effect size of 20% and a significance level of 0.05. The number of mice used, and the statistical methods applied were indicated in each figure legend. *P < 0.05, **P < 0.01, ***P < 0.001, ns: not significant.

### Supplementary information

Supplementary Information

## Data Availability

All data are available on request.
