# Supplementary material for: Nociceptive adenosine A2A receptor on trigeminal nerves orchestrates CGRP release to regulate the progression of oral squamous cell carcinoma
Source: Int J Oral Sci. 2024 Jun 18;16:46. doi: 10.1038/s41368-024-00308-w (PMC11183250; doi:10.1038/s41368-024-00308-w)
Supplement: Supplementary file 1 — Supplementary Information [file 41368_2024_308_MOESM1_ESM.docx]

**Supporting Information**

**Nociceptive Adenosine A_2A_ Receptor on Trigeminal Nerves Orchestrates CGRP Release to Regulate the Progression of Oral Squamous Cell Carcinoma**

Lanxin Jiang^1#^, Ying Zhou^1#^, Shijie Tang^1^, Dan Yang^1^, Yixin Zhang^1^, Jiuge Zhang^1^, Fan Yang^1^, Tong Zhou^1^, Xiaoqiang Xia^1^, Qianming Chen^2^, Lu Jiang^1^, Yuchen Jiang^1*^, Xiaodong Feng^1*^

^1^State Key Laboratory of Oral Diseases & National Center for Stomatology & National Clinical Research Center for Oral Diseases & Research Unit of Oral Carcinogenesis and Management, Chinese Academy of Medical Sciences, West China Hospital of Stomatology, Sichuan University, Chengdu, 610041, Sichuan, China

^2^Key Laboratory of Oral Biomedical Research of Zhejiang Province, Affiliated Stomatology Hospital, Zhejiang University School of Stomatology, Hangzhou, Zhejiang, 310006, China

Corresponding authors.

Email: [jiangyuchen16@126.com](mailto:jiangyuchen16@126.com) (Yuchen Jiang); [xiaodongfeng@scu.edu.cn](mailto:xiaodongfeng@scu.edu.cn) (Xiaodong Feng)


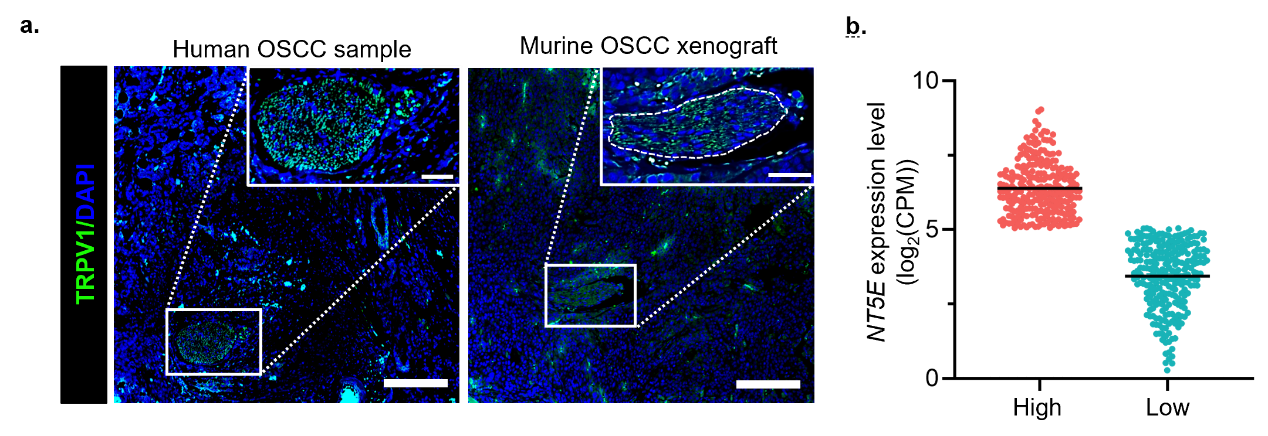


**Figure S1. Presence of nociceptive nerves in adenosine concentrated OSCC, related to Figure 1.** **a)** Immunofluorescent staining of TRPV1 positive neuron (green) and nuclei (blue) within human OSCC sample and mouse HSC3 cells xenograft. Scale bars, 200μm. Insets are magnification of boxed area. Scale bar, 50μm. The circled area is neuronal niche. **b)** Stratification of HNSC patients from TCGA database according to *NT5E* expression level. The ‘high’ and ‘low’ groups are defined as above or below the median of *NT5E* expression, *n* = 259 patients in ‘high’ or ‘low’ group.


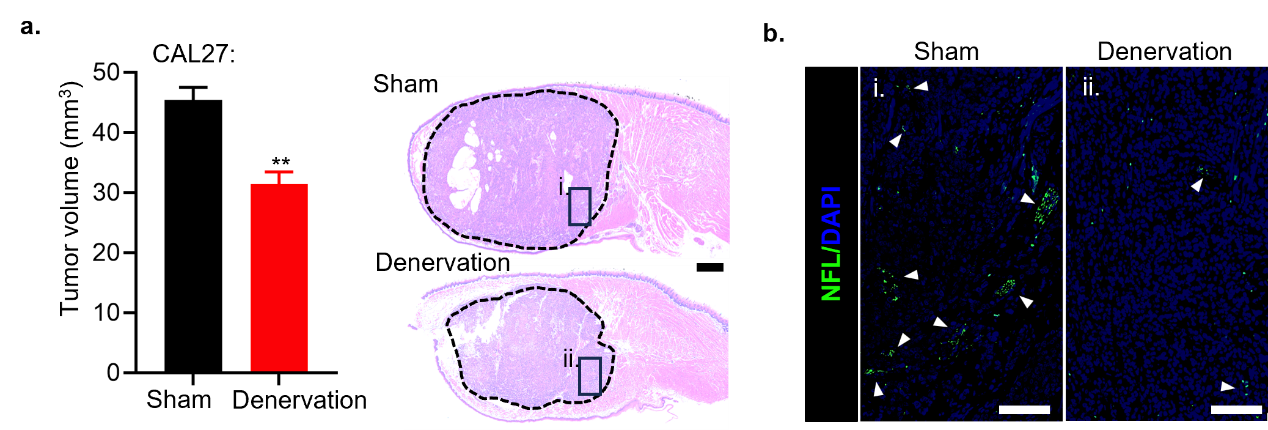


**Figure S2. Surgical denervation in CAL27 xenograft inhibited tumor growth, related to Figure 2. a)** Tumor volume of CAL27 xenografts in mice undergoing sham surgery or lingual nerve denervation, *n*=4 mice (Left panel). Representative HE images of CAL27 xenograft (circled) in mice undergoing sham surgery or lingual denervation (Right panel). Scale bar, 500µm. **b)** Immunofluorescent staining of neurofilament light chain (NFL) in framed area of **a)**. Scale bar, 100µm. Statistical analysis was conducted using unpaired Student’s t-test **(a)**.


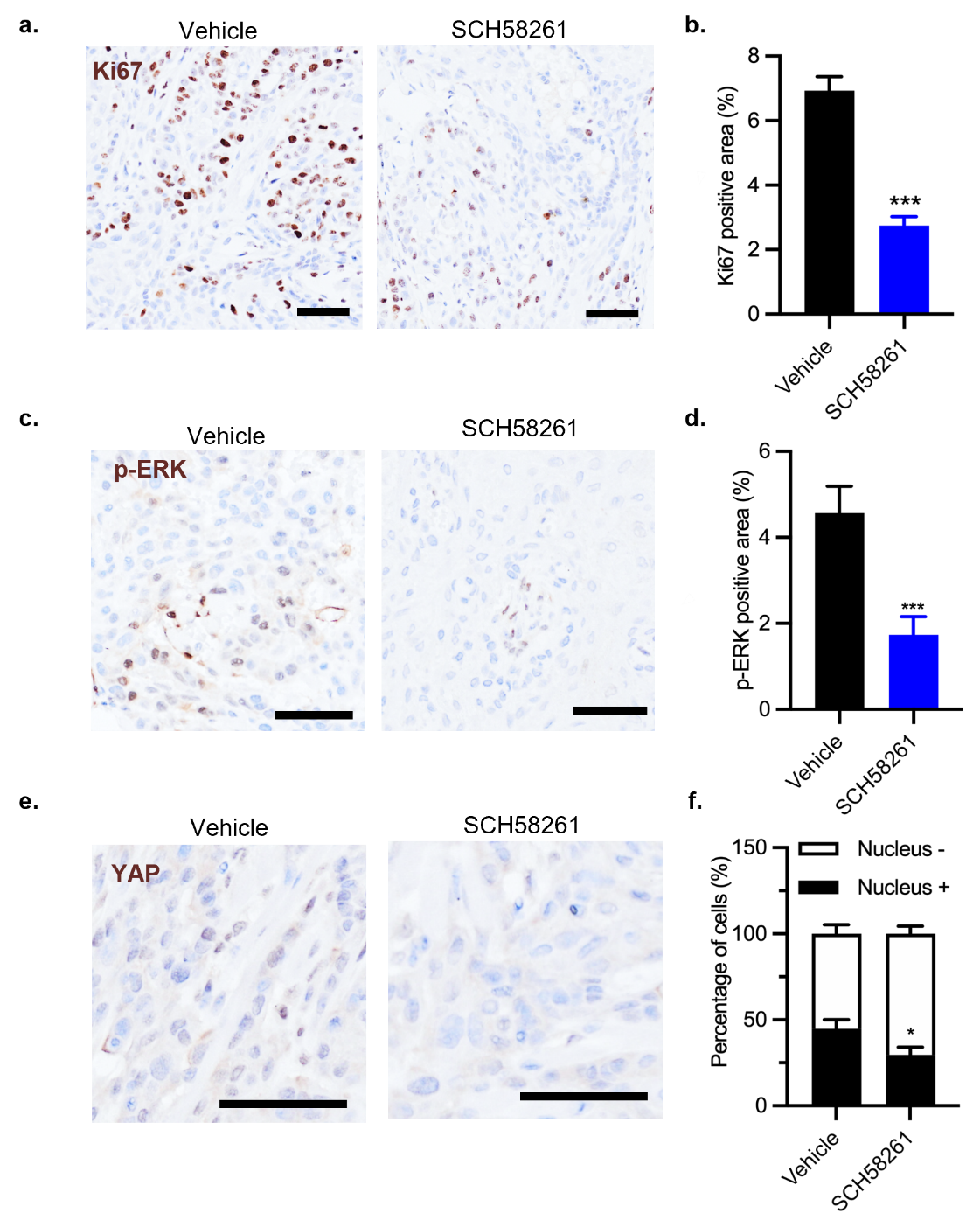


**Figure S3. SCH58261 induced a reduction in proliferative signatures in OSCC, related to Figure 3. a)**, Immunostaining of Ki67 in tumor xenograft in nontreated or treated group. Scale bars, 50μm. **b)**. Quantification of Ki67 positive area in tumor xenograft of nontreated or treated group, *n*=16 random fields from 4 mice. **c)**. Immunostaining of p-ERK in tumor xenograft of nontreated or treated group. Scale bars, 50μm. **d)**. Quantification of p-ERK positive area in tumor xenograft of nontreated or treated group, *n*=16 random fields from 4 mice. **e).** Immunostaining of YAP in tumor xenograft of nontreated or treated group. Scale bars, 50μm. **f)**. Quantification of percentage of YAP nuclear positive cells of nontreated or treated group, *n*=9 random fields from 3 mice. Statistical analysis was conducted using unpaired Student’s t-test **(b, d, f)**.

**Supplementary Table 1: Primers for real-time qPCR.**

| Gene | Primer | Sequence (5’-3’) |
| --- | --- | --- |
| *Adora2a* | Forward | GCCATCCCATTCGCCATCA |
|  | Reverse | GCAATAGCCAAGAGGCTGAAGA |
| *Calca* | Forward | TCCCCTTTCCTGGTTGTCAG |
|  | Reverse | GGCGAACTTCTTCTTCACTG |
| *Actb* | Forward | GAGACCTTCAACACCCCAGC |
|  | Reverse | ATGTCACGCACGATTTCCC |
